# Synthesis and Reactivity of Manganese Complexes Bearing Anionic PNP- and PCP-Type Pincer Ligands toward Nitrogen Fixation

**DOI:** 10.3390/molecules27072373

**Published:** 2022-04-06

**Authors:** Shogo Kuriyama, Shenglan Wei, Takeru Kato, Yoshiaki Nishibayashi

**Affiliations:** Department of Applied Chemistry, School of Engineering, The University of Tokyo, Hongo, Bunkyo-ku, Tokyo 113-8656, Japan; kuriyama2020@g.ecc.u-tokyo.ac.jp (S.K.); wei@crec.t.u-tokyo.ac.jp (S.W.); take0127kato@gmail.com (T.K.)

**Keywords:** manganese, nitrogen fixation, pincer ligand

## Abstract

A series of manganese complexes bearing an anionic pyrrole-based PNP-type pincer ligand and an anionic benzene-based PCP-type pincer ligand is synthesized and characterized. The reactivity of these complexes toward ammonia formation and silylamine formation from dinitrogen under mild conditions is evaluated to produce only stoichiometric amounts of ammonia and silylamine, probably because the manganese pincer complexes are unstable under reducing conditions.

## 1. Introduction

The production of ammonia from dinitrogen in the air, known as nitrogen fixation, has been an essential chemical process in industry and biology. The industrial nitrogen fixation, known as the Haber–Bosch process, requires harsh reaction conditions and dihydrogen derived from fossil fuels [1]. The development of new systems capable of fixing dinitrogen into ammonia efficiently under mild reaction conditions has remained a challenging goal.

Transition-metal catalyzed nitrogen fixation under mild reaction conditions has gained much attention in the last two decades [2,3,4,5,6,7,8,9,10,11]. Reduction of dinitrogen into ammonia [12,13,14,15,16,17,18,19,20,21,22,23,24,25] or its equivalents, such as hydrazine [26], silylamines [27,28,29,30,31,32,33,34,35,36,37,38,39,40,41,42,43,44], and borylamines [45], catalyzed by well-defined transition-metal complexes was found by several research groups. We have found that transition-metal complexes bearing anionic pincer ligands worked as highly active catalysts for nitrogen fixation. Recently, we have succeeded in the development of iron-, cobalt-, and vanadium-catalyzed formation of ammonia and hydrazine from dinitrogen under mild reaction conditions using the corresponding dinitrogen complexes bearing an anionic pyrrole-based PNP-type ligand as catalysts [46,47,48]. The PNP ligand was originally developed by groups of Gade, Mani, and Tonzetich [49,50,51]. More recently, we have reported that iron-dinitrogen complexes supported by a benzene-based PCP-type pincer ligand, one of the classical pincer ligands [52], exhibited a high catalytic activity for reduction of dinitrogen into ammonia and hydrazine [53]. In addition, a rhodium-dinitrogen complex supported by the same PNP-type ligand and an iridium-dinitrogen complex bearing the same benzene-based PCP-type ligand have been found to work as effective catalysts for the formation of silylamine from dinitrogen under mild reaction conditions [54,55].

Based on these backgrounds, we have focused on the development of manganese-catalyzed nitrogen fixation under mild reaction conditions. Manganese is the third most abundant transition-metal on the earth. However, successful examples of manganese-catalyzed nitrogen fixation under mild conditions are limited to the silylamine formation from dinitrogen [35,39]. Herein, we have prepared a series of manganese complexes bearing the anionic pyrrole-based PNP-type pincer ligand and the anionic benzene-based PCP-type pincer ligand to investigate their reactivity for the ammonia and silylamine formation under mild reaction conditions.

## 2. Result and Discussion

### 2.1. Synthesis and Characterization of Manganese Complexes

**Scheme 1:**
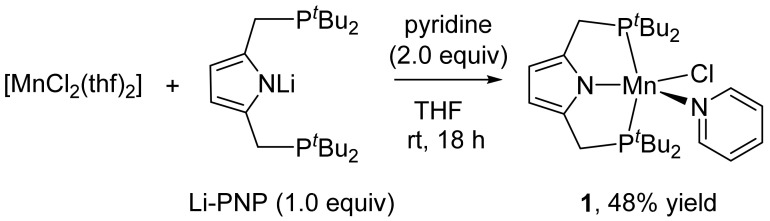
Synthesis of **1**.

**Figure 1:**
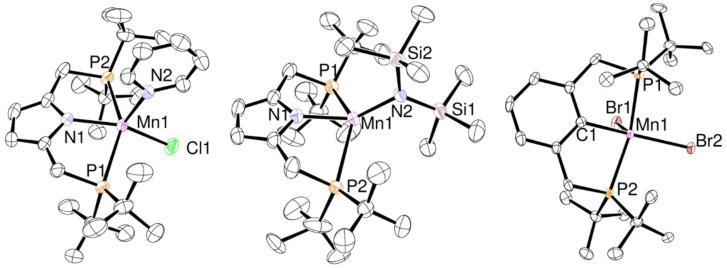
ORTEP drawings of **1** (**left**), **2** (**middle**), and **3** (**right**). Thermal ellipsoids are shown at the 50% probability level. Hydrogen atoms are omitted for clarity.

The reaction of [MnCl_2_(thf)_2_] (thf = tetrahydrofuran) with Li-PNP (PNP = 2,5-bis(di-*tert*-butylphosphinomethyl)pyrrolide) in the presence pyridine in THF at room temperature for 18 h gave a Mn(II) complex bearing the PNP-type pincer ligand [Mn(PNP)Cl(pyridine)] (**1**) in 48% yield (Scheme 1). The structure of **1** was confirmed by X-ray analysis, and an ORTEP drawing of **1** is shown in Figure 1. Table 1 presents selected bond lengths and angles of **1**. Complex **1** has a distorted square pyramidal geometry, where pyridine and chloride ligands are coordinated to the cobalt center supported by the PNP ligand. Complex **1** was paramagnetic with an effective solution magnetic moment of 5.8 ± 0.5 *μ*_B_ at room temperature by Evans’ method, in agreement with the theoretical value of *S* = 5/2 state (5.9 *μ*_B_). This result indicates that the manganese center of **1** is assigned as a d^5^ high-spin state. 

**Scheme 2:**
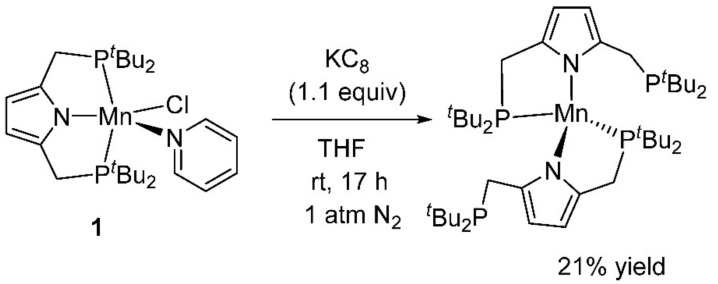
Reduction of **1**.

We attempted to prepare the corresponding manganese-dinitrogen complex by reduction of **1** with 1.1 equiv of KC_8_ in THF at room temperature under 1 atm N_2_ atmosphere (Scheme 2). However, no dinitrogen-coordinated species was observed by the IR spectrum. Instead, the recrystallization of the reaction mixture afforded a Mn(II) complex bearing the two PNP ligands [Mn(PNP)_2_] in 21% yield, which was previously reported by Tonzetich and coworkers [56].

**Scheme 3:**
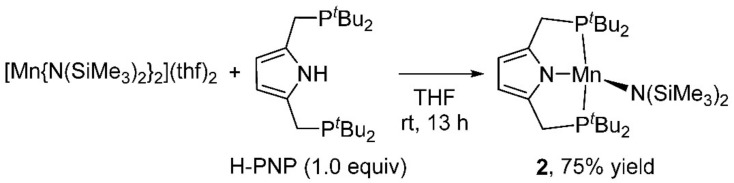
Synthesis of **2**.

The reaction of [Mn{N(SiMe_3_)_2_}_2_](thf)_2_ with H-PNP afforded a Mn(II)-amide complex bearing the PNP-type ligand [Mn(PNP)(N(SiMe_3_)_2_)] (**2**) in 75% yield (Scheme 3). An ORTEP drawing of **2** is shown in Figure 1, and bond lengths and angles of **2** are listed in Table 2. Complex **2** has a four-coordinated manganese center with a distorted tetrahedral structure. The Mn–P lengths of **2** (ca. 2.76 Å) are slightly longer than those of **1** (ca. 2.72 Å), while the P–Mn–P angle of **2** (132°) is smaller than that of **1** (147°). These structural differences between **1** and **2** may derive from the different coordination geometry around the cobalt centers. Complex **2** displayed a room-temperature magnetic moment of 6.0 ± 0.6 *μ*_B_ in solution, which indicates an *S* = 5/2 high-spin Mn(II) state.

**Scheme 4:**
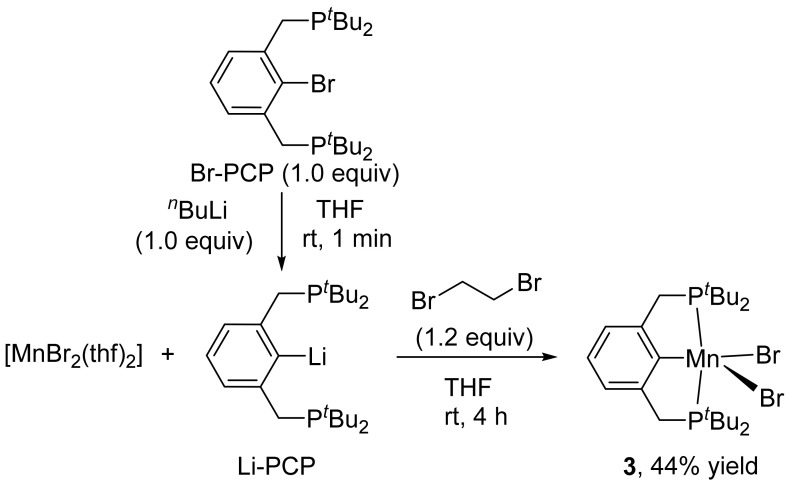
Synthesis of **3**.

The reaction of [MnBr_2_(thf)_2_] with Li-PCP (PCP = 2,6-bis(di-*tert*-butylphosphinomethyl)phenyl), which was generated in situ from Br-PCP with *n*-butyllithium, in the presence of 1,2-dibromoethane as an oxidant in THF at room temperature for 4 h gave a Mn(III) complex bearing the PCP-type ligand [Mn(PCP)Br_2_] (**3**) in 44% yield (Scheme 4). X-ray analysis revealed the molecular structure of **3**, and an ORTEP drawing and selected metric parameters are shown in Figure 1 and Table 3, respectively. The manganese center of **3** has a distorted square pyramidal geometry with two bromide ligands and the PCP ligand. One of the bromide ligands occupies the *trans* position to the C atom of the PCP ligand to make an equatorial plane, and the other is in the axial position. Shorter Mn–P bonds (ca. 2.49 Å) of **3** compared to those of **1** and **2** (2.72–2.76 Å) can be attributable to the six-membered backbone of the PCP ligand. The solution magnetic moment of **3** was 4.3 ± 0.4 *μ*_B_ at room temperature. Because this value is close to the theoretical value for an *S* = 2 high-spin state (4.9 *μ*_B_), **3** is assignable as an *S* = 2 state. Reported Mn(III)-aryl complexes exhibited effective magnetic moments between 4.8–5.3 *μ*_B_ [57,58,59].

### 2.2. Reactivity for Nitrogen Fixation

#### 2.2.1. Attempted Catalytic Ammonia Formation Using **1**–**3** as Catalysts

We investigated the catalytic reduction of dinitrogen into ammonia or hydrazine using the synthesized manganese complexes **1**–**3** as catalysts under the conditions previously applied for our group [46,47,48], which were initially reported by Peters [19]. Typical results are shown in Table 4. The reaction of an atmospheric pressure of dinitrogen with 40 equiv of KC_8_ as a reductant and 38 equiv of [H(OEt_2_)_2_]BAr^F^_4_ (Ar^F^ = 3,5-(CF_3_)_2_C_6_H_3_) as a proton source in the presence of a catalytic amount of **1** in Et_2_O was conducted at −78 °C for 1 h. As a result, neither ammonia nor hydrazine was obtained, while 7.8 equiv of dihydrogen based on the Mn atom were formed as a product (Table 4, entry 1). The use of **2** as a catalyst afforded 1.4 equiv of ammonia and 7.5 equiv of dihydrogen under the same reaction conditions (Table 4, entry 2), where 1 equiv of ammonia is considered to be derived from the N(SiMe_3_)_2_ ligand in **2**. We also attempted catalytic ammonia formation using complex **3** as a catalyst; however, no formation of ammonia nor hydrazine was observed. (Table 4, entry 3). These results indicate that the newly synthesized manganese complexes bearing the anionic PNP-type or PCP-type ligands did not work as catalysts for catalytic ammonia or hydrazine formation from dinitrogen.

#### 2.2.2. Attempted Catalytic Silylamine Formation Using **1**–**3** as Catalysts

We then tested **1**–**3** for their potential as catalysts for the formation of silylamine (N(SiMe_3_)_3_), which is easily converted to ammonia upon hydrolysis. Hence silylamine formation is regarded as an alternative nitrogen fixation process [8]. Typical results are shown in Table 5. The reaction of an atmospheric pressure of dinitrogen with 600 equiv of Na as a reductant and 600 equiv of Me_3_SiCl as a silylating reagent in the presence of **1** as a catalyst in THF was performed at room temperature for 20 h, followed by hydrolysis of the reaction mixture to convert silylamine to ammonia for quantification. This reaction afforded only 0.4 equiv of ammonia based on the Mn atom in the catalyst (Table 5, entry 1). When KC_8_ was used as a reductant in place of Na, 1.4 equiv of ammonia were obtained based on the Mn atom in the catalyst (Table 5, entry 2). The catalytic activity of **2** toward silylamine formation was also investigated in the same reaction conditions to give less than 2 equiv of ammonia (Table 5, entries 3 and 4). The use of **3** as a catalyst in the presence of Na or KC_8_ as a reductant afforded 2.5 or 2.2 equiv of ammonia based on the Mn atom in the catalyst, respectively, which are regarded as superstoichiometric amounts (Table 5, entries 5 and 6). These results suggest that these manganese complexes did not work as catalysts for silylamine formation under the current conditions. 

## 3. Materials and Methods

### 3.1. General Methods

^1^H NMR (400 MHz), ^13^C{^1^H} NMR (100 MHz), and ^31^P{^1^H} NMR (162 MHz) spectra were recorded on a JEOL ECS-400 spectrometer (Tokyo, Japan) or a JEOL ECZ-400S spectrometer in a suitable solvent, and spectra were referenced to residual solvent (^1^H, ^13^C{^1^H}) or external standard (^31^P{^1^H}: H_3_PO_4_). IR spectra were recorded on a JASCO FT/IR 4100 Fourier Transform infrared spectrometer (JASCO, Tokyo, Japan) or a Shimadzu IRSpirit spectrometer (Shimadzu, Kyoto, Japan). UV-vis absorption spectra were recorded on a Shimadzu UV-1850. Magnetic susceptibility was measured in C_6_D_6_ using the Evans method. Elemental analyses were performed at the Microanalytical Center of The University of Tokyo.

All manipulations were carried out under an atmosphere of nitrogen or argon by using standard Schlenk techniques or glovebox techniques, unless otherwise stated. Solvents were dried by general methods and degassed before use. Me_3_SiCl was distilled prior to use. Li-PNP [47], H-PNP [60,61,62], [MnCl_2_(thf)_2_] [63], [MnBr_2_(thf)_2_] [64], [Mn{N(SiMe_3_)_2_}](thf)_2_ [65], [H(OEt_2_)_2_]BAr^F^_4_ [20,66], and KC_8_ [67] were prepared according to the literature methods. All the other reagents were commercially available (reagent grade from TCI (Tokyo, Japan), FUJIFILM Wako Pure Chemical (Osaka, Japan), and Sigma-Aldrich (St. Louis, MO, USA)) and used as received.

### 3.2. Synthesis

#### 3.2.1. Preparation of PCP–Br

A mixture of 2-bromo-1,3-bis(bromomethyl)benzene (2.61 g, 7.61 mmol) and *^t^*Bu_2_PH (2.94 g, 20.1 mmol) in acetone (55 mL) was stirred at reflux temperature for 2 h. The mixture was cooled to room temperature, and then the solvent was removed in vacuo. The residue was washed with Et_2_O (8 mL, 3 times). NaOAc (5.0 g), Et_2_O (20 mL), and water (15 mL) were added to the white residue. The product was extracted by Et_2_O (15 mL, 3 times). The combined extracts were dried over anhydrous MgSO_4_, and then the mixture was filtered. The filtrate was evaporated to dryness to afford Br-PCP as a white solid (2.83 g, 5.98 mmol, 79%). ^1^H NMR (C_6_D_6_): δ 7.68 (d, *J* = 7.6 Hz, 2H), 7.04 (t, *J* = 7.6 Hz, 1H), 3.11 (s, 4H), 1.10 (d, *J* = 10.8 Hz, 36H). ^13^C{^1^H} NMR (C_6_D_6_): δ 141.9 (d, *J* = 13.4 Hz), 130.0 (d, *J* = 19.1 Hz), 128.5, 126.5, 32.0 (d, *J* = 23.9 Hz), 29.8 (d, *J* = 14.3 Hz), 29.7 (d, *J* = 24.8 Hz). ^31^P{^1^H} NMR (C_6_D_6_): δ 34.1 (s). Anal. Calcd. for C_24_H_43_BrP_2_: C, 60.88; H, 9.15. Found: C, 60.87, H, 9.03.

#### 3.2.2. Preparation of **1**

To a solution of Li-PNP (195 mg, 0.501 mmol) in THF (10 mL), pyridine (82 μL, 1.02 mmol) and [MnCl_2_(thf)_2_] (134 mg, 0.496 mmol) were added at room temperature. The reaction mixture was stirred at room temperature for 18 h, and the solvent was removed in vacuo. Et_2_O (10 mL) was added to the brown residue. The suspension was filtered through Celite, and the filter cake was washed with Et_2_O (3 mL, 4 times). The combined filtrate was concentrated into 5 mL. The resultant mixture was kept at −30 °C to give a yellow solid, which was collected by filtration, washed with a small amount of cold pentane, and dried in vacuo to afford **1** as a yellow solid (132 mg, 0.239 mmol, 48%). Single crystals of **1** suitable for X-ray crystallography were obtained as yellow crystals from Et_2_O at −30 °C. Magnetic susceptibility: *μ*_eff_ = 5.8 ± 0.5 *μ*_B_ in C_6_D_6_ at 298 K. Anal. Calcd. for C_27_H_47_ClN_2_P_2_Mn: C, 58.75; H, 8.58; N, 5.07. Found: C, 58.74; H, 8.57; N, 5.23.

#### 3.2.3. Preparation of **2**

To a solution of [Mn{N(SiMe_3_)_2_}](thf)_2_ (97.7 mg, 0.188 mmol) in THF (2 mL), a solution of H-PNP (68.3 mg, 0.178 mmol) in THF (2 mL) was added dropwise at room temperature. The reaction mixture was stirred at room temperature for 14 h, and then the solvent was removed in vacuo. Pentane (2 mL) was added to the pale-yellow residue. The suspension was filtered through Celite, and the filter cake was washed with pentane. The combined filtrate was concentrated into 1 mL. The resultant mixture was kept at −30 °C to give a pale orange solid, which was collected by decantation, washed with a small amount of cold pentane, and dried in vacuo to afford **2** as a pale orange solid (80.0 mg, 0.134 mmol, 75%). Single crystals of **2** suitable for X-ray crystallography were obtained as yellow crystals from pentane at −30 °C. Magnetic susceptibility: *μ*_eff_ = 6.0 ± 0.6 *μ*_B_ in C_6_D_6_ at 298 K. Anal. Calcd. for C_28_H_60_Si_2_N_2_P_2_Mn: C, 56.25; H, 10.12; N, 4.69. Found: C, 56.40; H, 10.20; N, 4.72.

#### 3.2.4. Preparation of **3**

To a solution of Br-PCP (142 mg, 0.300 mmol) in THF (5 mL), *^n^*BuLi (1.55 M in hexane, 195 μL, 0.302 mmol) was added at room temperature. The reaction mixture was stirred at room temperature for 1 min, then [MnBr_2_(thf)_2_] (107 mg, 0.298 mmol) and 1,2-dibromoethane (31 μL, 0.36 mmol) in THF (5 mL) were added to the mixture. The reaction mixture was stirred at room temperature for 4 h, and then the solvent was removed in vacuo. Benzene (5 mL) was added to the reddish-brown residue. The suspension was filtered through Celite, and the filter cake was washed with benzene (1 mL, 4 times). The combined filtrate was concentrated into 3 mL. Slow addition of hexane (12 mL) to the mixture afforded red crystals suitable for X-ray crystallography of **3**, which were collected by decantation and dried in vacuo to give **3·**0.5 C_6_H_6_ as a red crystalline solid (81.1 mg, 0.133 mmol, 44%). Magnetic susceptibility: *μ*_eff_ = 4.3 ± 0.4 *μ*_B_ in C_6_D_6_ at 298 K. Anal. Calcd. for C_27_H_46_Br_2_P_2_Mn (**3·**0.5 C_6_H_6_): C, 50.10; H, 7.16. Found: C, 50.77; H, 7.04. 

### 3.3. Reactivity for Nitrogen Fixation

#### 3.3.1. Attempted Catalytic Ammonia Formation Using **1**–**3** as Catalysts

A typical experimental procedure for reduction of dinitrogen to ammonia or hydrazine using **1** is described below. In a 50 mL Schlenk flask, **1** (5.2 mg, 10.0 μmol), KC_8_ (54.1 mg, 0.400 mmol) and [H(OEt_2_)_2_]BAr^F^_4_ (385 mg, 0.380 mmol) were placed. After the mixture was cooled to −78 °C, cold Et_2_O (5 mL) was added to the mixture. After the mixture was stirred at −78 °C for 1 h, the mixture was warmed to room temperature and further stirred at room temperature for 20 min. The amount of dihydrogen evolved in the reaction was determined by gas chromatography (GC) analyses. The reaction mixture was evaporated under reduced pressure, and the distillate was trapped in a dilute H_2_SO_4_ solution (0.5 M, 10 mL). Aqueous solution of potassium hydroxide (30 wt%, 5 mL) was added to the residue, and the mixture was distilled into the same dilute H_2_SO_4_ solution (0.5 M, 10 mL). The amount of NH_3_ present in H_2_SO_4_ solution was determined by the indophenol method [68]. The amount of NH_2_NH_2_ present in H_2_SO_4_ solution was determined by the *p*-(dimethylamino)benzaldehyde method [69]. 

#### 3.3.2. Attempted Catalytic Silylamine Formation Using **1**–**3** as Catalysts

A typical experimental procedure for reduction of dinitrogen to silylamine using **1** is described below. In a 50 mL Schlenk flask, **1** (2.4 mg, 5.0 μmol) and KC_8_ (406 mg, 3.00 mmol) were placed. After Et_2_O (6 mL) and Me_3_SiCl (380 μL, 3.00 mmol) were added to the mixture in the Schlenk, the mixture was stirred at room temperature for 40 h under N_2_ (1 atm). Dilute H_2_SO_4_ solution (0.5 M, 10 mL) was added to the mixture. The mixture was stirred at room temperature for 1 h. Aqueous solution of KOH (30 wt%, 5 mL) was added to the reaction mixture, and the mixture was distilled into another dilute H_2_SO_4_ solution (0.5 M, 10 mL). The amount of ammonia was determined by the indophenol method [68].

### 3.4. X-ray Diffraction

Crystallographic data of **1**–**3** are summarized in Table 6. Diffraction data for **1**–**3** were collected for the 2*θ* range of 4° to 55° at −100 °C (for **1** and **2**) or −180 °C (for **3**) on a Rigaku R-AXIS RAPID imaging plate area detector (Rigaku, Tokyo, Japan) with multi-layer mirror monochromated Mo-K*α* (*λ* = 0.71075 Å) radiation with VariMax optics. Intensity data were corrected for Lorentz and polarization effects and for empirical absorptions (ABSCOR [70] for **1**–**3**), while structure solutions and refinements were performed by using the *CrystalStructure* package [71]. The positions of non-hydrogen atoms were determined by direct methods (SHELXT version 2014/5 [72] for **1** and **3**; SHELXS version 2013/1 [73] for **2**) and subsequent Fourier syntheses (SHELXL [74] version 2016/6) and were refined on *F_o_*^2^ using all unique reflections by full-matrix least-squares with anisotropic thermal parameters. All the hydrogen atoms were placed at the calculated positions with fixed isotropic parameters. Crystal of **1** and **3** contains an inverted structure as a disorder, which was solved as merohedral twins. Crystal of **2** contains heavy disorders among a *tert*-butyl group, which were solved as disorders in a ratio of 0.52:0.48 for C(21A)–C(22A) and C(21B)–C(22B). All the hydrogen atoms attached to these carbon atoms could not be located, causing checkCIF/PLATON report Alert level B (PLAT043_ALERT_1_B Calculated and Reported Mol. Weight Differ by 6.06).

## 4. Conclusions

In the current work, three manganese complexes bearing anionic PNP- and PCP-type pincer ligands were synthesized and fully characterized. Their catalytic activities for nitrogen fixation, such as ammonia and silylamine formation, were extensively studied. As a result, unfortunately, these manganese complexes did not promote the catalytic formation of ammonia or silylamine from nitrogen gas under mild reaction conditions. These results are in contrast to the previous results that vanadium, iron, cobalt, rhodium, and iridium complexes bearing the identical anionic pincer ligands worked as catalysts for ammonia formation or silylamine formation under mild reaction conditions [46,47,48,54,55]. Currently, we consider that the manganese pincer complexes are unstable under reducing conditions to cause catalyst decomposition. We hope the present result will help researchers to design nitrogen fixation systems using transition-metal complexes as catalysts. 

## Figures and Tables

**Table 1:** Selected Bond Lengths (Å) and Angles (deg) for **1**.

Mn(1)–P(1)	2.7189(8)	Mn(1)–P(2)	2.7275(7)
Mn(1)–N(1)	2.127(2)	Mn(1)–N(2)	2.188(2)
Mn(1)–Cl(1)	2.3716(10)		
			
P(1)–Mn(1)–P(2)	147.46(3)	N(1)–Mn(1)–N(2)	107.34(8)
N(1)–Mn(1)–Cl(1)	152.86(6)		

**Table 2:** Selected Bond Lengths (Å) and Angles (deg) for **2**.

Mn(1)–P(1)	2.7618(8)	Mn(1)–P(2)	2.7539(7)
Mn(1)–N(1)	2.0549(18)	Mn(1)–N(2)	2.0215(19)
			
P(1)–Mn(1)–P(2)	132.94(2)	N(1)–Mn(1)–N(2)	139.15(6)

**Table 3:** Selected Bond Lengths (Å) and Angles (deg) for **3**.

Mn(1)–P(1)	2.4792(17)	Mn(1)–P(2)	2.4865(17)
Mn(1)–Br(1)	2.5208(9)	Mn(1)–Br(2)	2.4513(9)
Mn(1)–C(1)	2.071(5)		
			
P(1)–Mn(1)–P(2)	144.70(6)	C(1)–Mn(1)–Br(1)	95.46(14)
C(1)–Mn(1)–Br2(1)	163.25(14)		

**Table 4:**
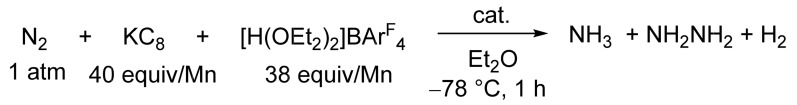
Attempted catalytic reduction of dinitrogen into ammonia or hydrazine using **1**–**3** at −78 °C *^a^*.

Entry	cat.	NH_3_ (Equiv) *^b^*	NH_2_NH_2_ (Equiv) *^b^*	H_2_ (Equiv) *^b^*
1	**1**	0	0	7.8
2	**2**	1.4	0	7.5
3	**3**	0	0	7.0

*^a^* A mixture of cat. (1 equiv), KC_8_ (40 equiv) and [H(OEt_2_)_2_][BAr^F^_4_] (38 equiv) was stirred in Et_2_O at −78 °C for 1 h under 1 atm of dinitrogen and then at room temperature for 20 min. *^b^* Based on the manganese atom in the catalyst.

**Table 5:**
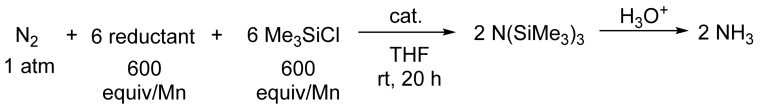
Attempted catalytic reduction of dinitrogen into silylamine using **1**–**3** at rt *^a^*.

Entry	cat.	Reductant	NH_3_ (Equiv) *^b^*
1	**1**	Na	0.4
2	**1**	KC_8_	1.5
3	**2**	Na	0.8
4	**2**	KC_8_	1.4
5	**3**	Na	2.5
6	**3**	KC_8_	2.2

*^a^* A mixture of cat. (1 equiv), KC_8_ (600 equiv), and Me_3_SiCl (600 equiv) was stirred in THF at room temperature for 20 h under 1 atm of dinitrogen. Silylamine was quantified as ammonia after acid hydrolysis of the reaction mixture. *^b^* Based on the manganese atom in the catalyst.

**Table 6:** X-ray crystallographic data for **1**–**3**.

Compound	1	2	3
chemical formula	C_27_H_47_ClMnN_2_P_2_	C_28_H_60_MnN_2_P_2_Si_2_	C_24_H_43_Br_2_MnP_2_
CCDC number	2149812	2149810	2149811
formula weight	552.02	597.85	608.30
dimensions of crystals, mm^3^	0.500 × 0.300 × 0.200	0.300 × 0.300 × 0.300	0.150 × 0.050 × 0.050
crystal color, habit	orange, block	colorless, chunk	red, block
crystal system	orthorhombic	monoclinic	orthorhombic
space group	*P*na2_1_ (#33)	*P*2_1_/n (#14)	*P*na2_1_ (#33)
*a*, Å	19.9909(11)	10.6261(3)	12.2701(4)
*b*, Å	19.141(2)	24.7389(7)	15.6675(5)
*c*, Å	7.849(3)	14.0062(4)	14.2993(4)
*α*, deg	90	90	90
*β*, deg	90	107.034(8)	90
*γ*, deg	90	90	90
*V*, Å^3^	3003.4(10)	3520.4(2)	2748.92(15)
*Z*	4	4	4
*ρ*_calcd_, g·cm^−3^	1.221	1.128	1.470
*F*(000)	1180.00	1300.00	1248.00
*μ*, cm^−1^	6.519	5.518	35.217
trans. factors range	0.647–0.878	0.728–0.847	0.528–0.839
no. reflections measured	28090	33691	25377
no. unique reflections	6867	8055	5991
no. parameters refined	0.0398	0.0457	0.0835
*R*1 (*I* > 2σ(*I*)) *^a^*	0.0322	0.0396	0.0386
*wR2* (all data) *^b^*	0.0645	0.0863	0.0652
GOF *^c^*	1.042	1.005	0.975
flack parameter	0.000		0.000
max diff peak/hole, e Å^−3^	0.30/−0.16	0.47/−0.34	0.73/−0.35

*^a^ R*1 = Σ||*F*_o_|−|*F*_c_||/Σ|*F*_o_|. *^b^ wR*2 = [Σ*w*(*F*_o_^2^−*F*_c_^2^)^2^/Σ*w*(*F*_o_^2^)^2^]^1/2^, *w* = 1/[*σ*^2^(*F*_o_^2^) + (*qP*)^2^ + *rP*], P = (Max(*F*_o_^2^, 0) + 2*F*_c_^2^)/3 [*q* = 0.0265 (**1**), 0 (**2**), 0.0235 (**3**); *r* = 0.6590 (**1**), 3.8000 (**2**), 0 (**3**)]. *^c^* GOF = [Σ*w*(*F*_o_^2^−*F*_c_^2^)^2^/(*N*_o_−*N*_params_)]^1/2^.

## Data Availability

The crystallographic data are available from the Cambridge Crystallographic Data Centre (CCDC).

## References

[1] Liu H. (2013). Ammonia Synthesis Catalysts: Innovation and Practice.

[2] Masero F., Perrin M.A., Dey S., Mougel V. (2021). Dinitrogen Fixation: Rationalizing Strategies Utilizing Molecular Complexes. Chem.-Eur. J..

[3] Tanabe Y., Nishibayashi Y. (2021). Comprehensive Insights into Synthetic Nitrogen Fixation Assisted by Molecular Catalysts under Ambient or Mild Conditions. Chem. Soc. Rev..

[4] Kuriyama S., Nishibayashi Y. (2021). Development of Catalytic Nitrogen Fixation Using Transition Metal Complexes Not Relevant to Nitrogenases. Tetrahedron.

[5] Ashida Y., Nishibayashi Y. (2021). Catalytic Conversion of Nitrogen Molecule into Ammonia Using Molybdenum Complexes under Ambient Reaction Conditions. Chem. Commun..

[6] Chalkley M.J., Drover M.W., Peters J.C. (2020). Catalytic N_2_-to-NH_3_ (or -N_2_H_4_) Conversion by Well-Defined Molecular Coordination Complexes. Chem. Rev..

[7] Kim S., Loose F., Chirik P.J. (2020). Beyond Ammonia: Nitrogen–Element Bond Forming Reactions with Coordinated Dinitrogen. Chem. Rev..

[8] Tanabe Y., Nishibayashi Y. (2019). Recent Advances in Catalytic Silylation of Dinitrogen Using Transition Metal Complexes. Coord. Chem. Rev..

[9] Stucke N., Flöser B.M., Weyrich T., Tuczek F. (2018). Nitrogen Fixation Catalyzed by Transition Metal Complexes: Recent Developments. Eur. J. Inorg. Chem..

[10] Nishibayashi Y. (2018). Development of Catalytic Nitrogen Fixation Using Transition Metal–Dinitrogen Complexes under Mild Reaction Conditions. Dalton Trans..

[11] Burford R.J., Fryzuk M.D. (2017). Examining the Relationship between Coordination Mode and Reactivity of Dinitrogen. Nat. Rev. Chem..

[12] Doyle L.R., Wooles A.J., Jenkins L.C., Tuna F., McInnes E.J.L., Liddle S.T. (2018). Catalytic Dinitrogen Reduction to Ammonia at a Triamidoamine–Titanium Complex. Angew. Chem. Int. Ed..

[13] Ashida Y., Egi A., Arashiba K., Tanaka H., Mitsumoto T., Kuriyama S., Yoshizawa K., Nishibayashi Y. (2022). Catalytic Reduction of Dinitrogen into Ammonia and Hydrazine Using Chromium Complexes Bearing PCP-Type Pincer Ligand. Chem.-Eur. J..

[14] Yandulov D.V., Schrock R.R. (2003). Catalytic Reduction of Dinitrogen to Ammonia at a Single Molybdenum Center. Science.

[15] Arashiba K., Miyake Y., Nishibayashi Y. (2011). A Molybdenum Complex Bearing PNP-Type Pincer Ligands Leads to the Catalytic Reduction of Dinitrogen into Ammonia. Nat. Chem..

[16] Arashiba K., Eizawa A., Tanaka H., Nakajima K., Yoshizawa K., Nishibayashi Y. (2017). Catalytic Nitrogen Fixation via Direct Cleavage of Nitrogen–Nitrogen Triple Bond of Molecular Dinitrogen under Ambient Reaction Conditions. Bull. Chem. Soc. Jpn..

[17] Ashida Y., Arashiba K., Nakajima K., Nishibayashi Y. (2019). Molybdenum-Catalysed Ammonia Production with Samarium Diiodide and Alcohols or Water. Nature.

[18] Meng F., Kuriyama S., Tanaka H., Egi A., Yoshizawa K., Nishibayashi Y. (2021). Ammonia Formation Catalyzed by a Dinitrogen-Bridged Dirhenium Complex Bearing PNP-Pincer Ligands under Mild Reaction Conditions. Angew. Chem. Int. Ed..

[19] Anderson J.S., Rittle J., Peters J.C. (2013). Catalytic Conversion of Nitrogen to Ammonia by an Iron Model Complex. Nature.

[20] Del Castillo T.J., Thompson N.B., Peters J.C. (2016). A Synthetic Single-Site Fe Nitrogenase: High Turnover, Freeze-Quench ^57^Fe Mössbauer Data, and a Hydride Resting State. J. Am. Chem. Soc..

[21] Chalkley M.J., Del Castillo T.J., Matson B.D., Roddy J.P., Peters J.C. (2017). Catalytic N_2_-to-NH_3_ Conversion by Fe at Lower Driving Force: A Proposed Role for Metallocene-Mediated PCET. ACS Cent. Sci..

[22] Buscagan T.M., Oyala P.H., Peters J.C. (2017). N_2_-to-NH_3_ Conversion by a Triphos-Iron Catalyst and Enhanced Turnover under Photolysis. Angew. Chem. Int. Ed..

[23] Del Castillo T.J., Thompson N.B., Suess D.L.M., Ung G., Peters J.C. (2015). Evaluating Molecular Cobalt Complexes for the Conversion of N_2_ to NH_3_. Inorg. Chem..

[24] Dorantes M.J., Moore J.T., Bill E., Mienert B., Lu C.C. (2020). Bimetallic Iron–Tin Catalyst for N_2_ to NH_3_ and a Silyldiazenido Model Intermediate. Chem. Commun..

[25] Fajardo J., Peters J.C. (2017). Catalytic Nitrogen-to-Ammonia Conversion by Osmium and Ruthenium Complexes. J. Am. Chem. Soc..

[26] Hill P.J., Doyle L.R., Crawford A.D., Myers W.K., Ashley A.E. (2016). Selective Catalytic Reduction of N_2_ to N_2_H_4_ by a Simple Fe Complex. J. Am. Chem. Soc..

[27] Shiina K. (1972). Reductive Silylation of Molecular Nitrogen via Fixation to Tris(Trialkylsilyl)Amine. J. Am. Chem. Soc..

[28] Ghana P., van Krüchten F.D., Spaniol T.P., van Leusen J., Kögerler P., Okuda J. (2019). Conversion of Dinitrogen to Tris(Trimethylsilyl)Amine Catalyzed by Titanium Triamido-Amine Complexes. Chem. Commun..

[29] Imayoshi R., Nakajima K., Nishibayashi Y. (2017). Vanadium-Catalyzed Reduction of Molecular Dinitrogen into Silylamine under Ambient Reaction Conditions. Chem. Lett..

[30] Kendall A.J., Johnson S.I., Bullock R.M., Mock M.T. (2018). Catalytic Silylation of N_2_ and Synthesis of NH_3_ and N_2_H_4_ by Net Hydrogen Atom Transfer Reactions Using a Chromium P_4_ Macrocycle. J. Am. Chem. Soc..

[31] Yin J., Li J., Wang G.-X., Yin Z.-B., Zhang W.-X., Xi Z. (2019). Dinitrogen Functionalization Affording Chromium Hydrazido Complex. J. Am. Chem. Soc..

[32] Komori K., Oshita H., Yasushi M., Hidai M. (1989). Catalytic Conversion of Molecular Nitrogen into Silylamines Using Molybdenum and Tungsten Dinitrogen Complexes. J. Am. Chem. Soc..

[33] Tanaka H., Sasada A., Kouno T., Yuki M., Miyake Y., Nakanishi H., Nishibayashi Y., Yoshizawa K. (2011). Molybdenum-Catalyzed Transformation of Molecular Dinitrogen into Silylamine: Experimental and DFT Study on the Remarkable Role of Ferrocenyldiphosphine Ligands. J. Am. Chem. Soc..

[34] Imayoshi R., Tanaka H., Matsuo Y., Yuki M., Nakajima K., Yoshizawa K., Nishibayashi Y. (2015). Cobalt-Catalyzed Transformation of Molecular Dinitrogen into Silylamine under Ambient Reaction Conditions. Chem.-Eur. J..

[35] Eaton M.C., Knight B.J., Catalano V.J., Murray L.J. (2020). Evaluating Metal Ion Identity on Catalytic Silylation of Dinitrogen Using a Series of Trimetallic Complexes. Eur. J. Inorg. Chem..

[36] Yuki M., Tanaka H., Sasaki K., Miyake Y., Yoshizawa K., Nishibayashi Y. (2012). Iron-Catalysed Transformation of Molecular Dinitrogen into Silylamine under Ambient Conditions. Nat. Commun..

[37] Prokopchuk D.E., Wiedner E.S., Walter E.D., Popescu C.V., Piro N.A., Kassel W.S., Bullock R.M., Mock M.T. (2017). Catalytic N_2_ Reduction to Silylamines and Thermodynamics of N_2_ Binding at Square Planar Fe. J. Am. Chem. Soc..

[38] Araake R., Sakadani K., Tada M., Sakai Y., Ohki Y. (2017). [Fe_4_] and [Fe_6_] Hydride Clusters Supported by Phosphines: Synthesis, Characterization, and Application in N_2_ Reduction. J. Am. Chem. Soc..

[39] Ohki Y., Araki Y., Tada M., Sakai Y. (2017). Synthesis and Characterization of Bioinspired [Mo_2_Fe_2_]–Hydride Cluster Complexes and Their Application in the Catalytic Silylation of N_2_. Chem.-Eur. J..

[40] Arata S., Sunada Y. (2019). An Isolable Iron(ii) Bis(Supersilyl) Complex as an Effective Catalyst for Reduction Reactions. Dalton Trans..

[41] Siedschlag R.B., Bernales V., Vogiatzis K.D., Planas N., Clouston L.J., Bill E., Gagliardi L., Lu C.C. (2015). Catalytic Silylation of Dinitrogen with a Dicobalt Complex. J. Am. Chem. Soc..

[42] Suzuki T., Fujimoto K., Takemoto Y., Wasada-Tsutsui Y., Ozawa T., Inomata T., Fryzuk M.D., Masuda H. (2018). Efficient Catalytic Conversion of Dinitrogen to N(SiMe_3_)_3_ Using a Homogeneous Mononuclear Cobalt Complex. ACS Catal..

[43] Gao Y., Li G., Deng L. (2018). Bis(Dinitrogen)Cobalt(−1) Complexes with NHC Ligation: Synthesis, Characterization, and Their Dinitrogen Functionalization Reactions Affording Side-on Bound Diazene Complexes. J. Am. Chem. Soc..

[44] Arnold P.L., Ochiai T., Lam F.Y.T., Kelly R.P., Seymour M.L., Maron L. (2020). Metallacyclic Actinide Catalysts for Dinitrogen Conversion to Ammonia and Secondary Amines. Nat. Chem..

[45] Bennaamane S., Espada M.F., Mulas A., Personeni T., Saffon-Merceron N., Fustier-Boutignon M., Bucher C., Mézailles N. (2021). Catalytic Reduction of N_2_ to Borylamine at a Molybdenum Complex. Angew. Chem. Int. Ed..

[46] Sekiguchi Y., Arashiba K., Tanaka H., Eizawa A., Nakajima K., Yoshizawa K., Nishibayashi Y. (2018). Catalytic Reduction of Molecular Dinitrogen to Ammonia and Hydrazine Using Vanadium Complexes. Angew. Chem. Int. Ed..

[47] Kuriyama S., Arashiba K., Nakajima K., Matsuo Y., Tanaka H., Ishii K., Yoshizawa K., Nishibayashi Y. (2016). Catalytic Transformation of Dinitrogen into Ammonia and Hydrazine by Iron-Dinitrogen Complexes Bearing Pincer Ligand. Nat. Commun..

[48] Kuriyama S., Arashiba K., Tanaka H., Matsuo Y., Nakajima K., Yoshizawa K., Nishibayashi Y. (2016). Direct Transformation of Molecular Dinitrogen into Ammonia Catalyzed by Cobalt Dinitrogen Complexes Bearing Anionic PNP Pincer Ligands. Angew. Chem. Int. Ed..

[49] Grüger N., Wadepohl H., Gade L.H. (2012). A Readily Accessible PNP Pincer Ligand with a Pyrrole Backbone and Its Ni^I/II^ Chemistry. Dalton Trans..

[50] Kumar S., Mani G., Mondal S., Chattaraj P.K. (2012). Pyrrole-Based New Diphosphines: Pd and Ni Complexes Bearing the PNP Pincer Ligand. Inorg. Chem..

[51] Venkanna G.T., Ramos T.V.M., Arman H.D., Tonzetich Z.J. (2012). Nickel (II) Complexes Containing a Pyrrole–Diphosphine Pincer Ligand. Inorg. Chem..

[52] Moulton C.J., Shaw B.L. (1976). Transition Metal–Carbon Bonds. Part XLII. Complexes of Nickel, Palladium, Platinum, Rhodium and Iridium with the Tridentate Ligand 2,6-Bis[(Di-t-Butylphosphino)Methyl]Phenyl. J. Chem. Soc. Dalton Trans..

[53] Kuriyama S., Kato T., Tanaka H., Konomi A., Yoshizawa K., Nishibayashi Y. (2022). Catalytic Reduction of Dinitrogen to Ammonia and Hydrazine Using Iron–Dinitrogen Complexes Bearing Anionic Benzene-Based PCP-Type Pincer Ligands. Bull. Chem. Soc. Jpn..

[54] Kawakami R., Kuriyama S., Tanaka H., Arashiba K., Konomi A., Nakajima K., Yoshizawa K., Nishibayashi Y. (2019). Catalytic Reduction of Dinitrogen to Tris(Trimethylsilyl)Amine Using Rhodium Complexes with a Pyrrole-Based PNP-Type Pincer Ligand. Chem. Commun..

[55] Kawakami R., Kuriyama S., Tanaka H., Konomi A., Yoshizawa K., Nishibayashi Y. (2020). Iridium-Catalyzed Formation of Silylamine from Dinitrogen under Ambient Reaction Conditions. Chem. Lett..

[56] Narro A.L., Arman H.D., Tonzetich Z.J. (2019). Manganese Chemistry of Anionic Pyrrole-Based Pincer Ligands. Organometallics.

[57] Sarbajna A., He Y.-T., Dinh M.H., Gladkovskaya O., Rahaman S.M.W., Karimata A., Khaskin E., Lapointe S., Fayzullin R.R., Khusnutdinova J.R. (2019). Aryl–X Bond-Forming Reductive Elimination from High-Valent Mn–Aryl Complexes. Organometallics.

[58] Morris R.J., Girolami G.S. (1991). High-Valent Organomanganese Chemistry. 2. Synthesis and Characterization of Manganese(III) Aryls. Organometallics.

[59] Dey K., De R.L. (1977). Organometallic Derivatives of Cobalt(III), Chromium(III) and Manganese(III) Complexes of Schiff Bases. J. Inorg. Nucl. Chem..

[60] Kreye M., Freytag M., Jones P.G., Williard P.G., Bernskoetter W.H., Walter M.D. (2015). Homolytic H_2_ Cleavage by a Mercury-Bridged Ni(i) Pincer Complex [{(PNP)Ni}_2_{μ-Hg}]. Chem. Commun..

[61] Venkanna G.T., Arman H.D., Tonzetich Z.J. (2014). Catalytic C–S Cross-Coupling Reactions Employing Ni Complexes of Pyrrole-Based Pincer Ligands. ACS Catal..

[62] Levine D.S., Tilley T.D., Andersen R.A. (2015). C–H Bond Activations by Monoanionic, PNP-Supported Scandium Dialkyl Complexes. Organometallics.

[63] Fowles G.W.A., Rice D.A., Walton R.A. (1969). The Donor Properties of Simple Ethers—II[1]: Complexes of Manganese(II), Iron(II), Cobalt(II) and Nickel(II) Halides with Tetrahydrofuran and 1,2-Dimethoxyethane. J. Inorg. Nucl. Chem..

[64] Murray B.D., Power P.P. (1984). Monomeric Manganese(II) Alkoxides: Syntheses and x-Ray Crystal Structures of Novel Three- and Four-Coordinate Manganese Complexes of the Tri-Tert-Butylmethoxide Ligand. J. Am. Chem. Soc..

[65] Bradley D.C., Hursthouse M.B., Ibrahim A.A., Malik K.M.A., Motevalli M., Möseler R., Powell H., Runnacles J.D., Sullivan A.C. (1990). Synthesis and Chemistry of the Bis(Trimethylsilyl)Amido Bis-Tetrahydrofuranates of the Group 2 Metals Magnesium, Calcium, Strontium and Barium. X-ray Crystal Structures of Mg[N(SiMe_3_)_2_]_2_·2THF and Related Mn[N(SiMe_3_)_2_]_2_·2THF. Polyhedron.

[66] Brookhart M., Grant B., Volpe A.F. (1992). [(3,5-(CF_3_)_2_C_6_H_3_)_4_B]^-^[H(OEt_2_)_2_]^+^: A Convenient Reagent for Generation and Stabilization of Cationic, Highly Electrophilic Organometallic Complexes. Organometallics.

[67] Weitz I.S., Rabinovitz M. (1993). The Application of C8K for Organic Synthesis: Reduction of Substituted Naphthalenes. J. Chem. Soc. Perkin 1.

[68] Weatherburn M.W. (1967). Phenol-Hypochlorite Reaction for Determination of Ammonia. Anal. Chem..

[69] Watt G.W., Chrisp J.D. (1952). Spectrophotometric Method for Determination of Hydrazine. Anal. Chem..

[70] Higashi T. (1995). ABSCOR: Program for Absorption Correction.

[71] (2000–2018). CrystalStructure.

[72] Sheldrick G.M. (2014). SHELXT: Integrating space group determination and structure solution. Acta Crystallogr. Sect. A Found. Adv..

[73] Sheldrick G.M. (2008). A short history of SHELX. Acta Crystallogr. Sect. A Found. Crystallogr..

[74] Sheldrick G.M. (2015). Crystal structure refinement with SHELXL. Acta Crystallogr. Sect. C Struct. Chem..

